# Operative Treatment of Cervical Myelopathy: Cervical Laminoplasty

**DOI:** 10.1155/2012/508534

**Published:** 2012-05-28

**Authors:** Brett A. Braly, David Lunardini, Chris Cornett, William F. Donaldson

**Affiliations:** ^1^Univeristy of Pittsburgh Medical Center, Pittsburgh, PA, USA; ^2^University of Nebraska Medical Center, Omaha, NE, USA

## Abstract

Cervical spondylotic myelopathy (CSM) is a degenerative process which may result in clinical signs and symptoms which require surgical intervention. Many treatment options have been proposed with various degrees of technical difficulty and technique sensitive benefits. We review laminoplasty as a motion-sparing posterior decompressive method. Current literature supports the use of laminoplasty for indicated decompression. We also decribe our surgical technique for an open-door, or “hinged”, laminoplasty.

## 1. Introduction

 Cervical spondylotic myelopathy (CSM) is the natural result of degenerative compression on the cervical spinal cord. The result may be a progressive and stepwise deterioration of neurological function in patients. The chronic debilitating nature of this process justifies surgical decompression. Posterior decompression has been described as a treatment for CSM since the 1940s. Laminectomy was the initial surgical option used. The decompression was performed by rongeurs. However, the insertion of the rongeur in an already limited space available for the cord led often to a decrease in neurological function postoperatively [1–3]. Even with modern approaches to laminectomy using high speed burs, development of postoperative instability has led surgeons to explore more efficacious ways of decompression.

 In 1977, Hirabayashi and Satomi published their results on multisegment decompression by means of an open-door laminoplasty [4]. This technique allows for adequate posterior decompression of the spinal cord while retaining the posterior elements. This avoids the postoperative instability seen with laminectomy as well as the stiffness and risks of posterior cervical fusion. Additionally, motion is spared due to the absence of a fusion. There have since been multiple techniques for performing a cervical laminoplasty described with supporting literature [4–8]. These techniques include the expansive “open door,” a midline “French Door,” *En Bloc* resection, spinous process splitting, and Z-Plasty [4, 9]. Outcome studies have supported laminoplasty as a valid treatment for CSM however, no definitive literature shows its superiority to laminectomy in conjunction with a posterior cervical fusion. All surgical strategies appear to be equal in yielding neurologic outcomes, though differences are found in complication reports.

 Patient selection is crucial prior to proceeding with cervical laminoplasty. Special attention must be paid to sagittal alignment for optimal outcomes. Laminoplasty is ideal for multilevel stenosis (AP canal diameter < 13 mm) due to spondylosis or ossification of the posterior longitudinal ligament (OPLL) [10].

 Posterior cervical decompression, either by laminoplasty or laminectomy/fusion, carries inherent risks. Laminoplasty has been associated with postoperative C5 palsy, persistent axial neck pain, and some loss of range of motion [10–13].

## 2. Patient Selection, Indications, and Contraindications

**Figure 1:**
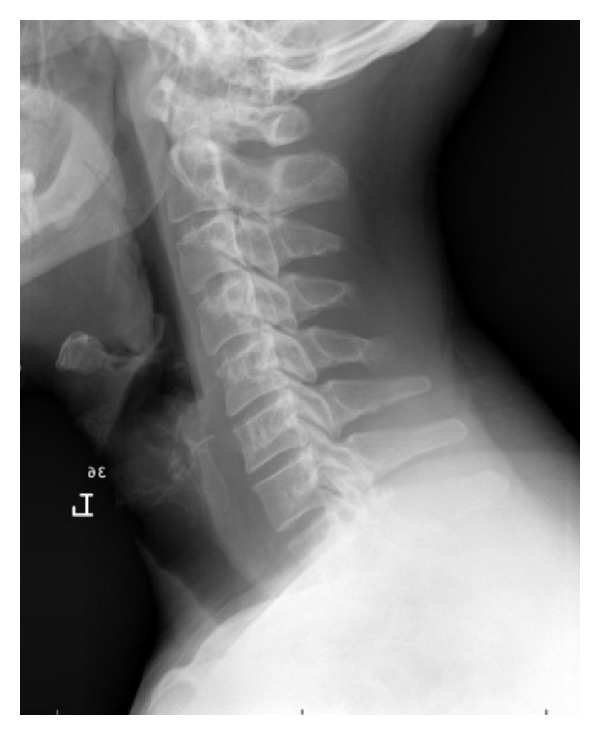
Lateral radiograph of the cervical spine in a patient who underwent laminoplasty. Note that there is an overall lordotic alignment which will allow for posterior drift once a posterior decompression is performed.

**Figure 2:**
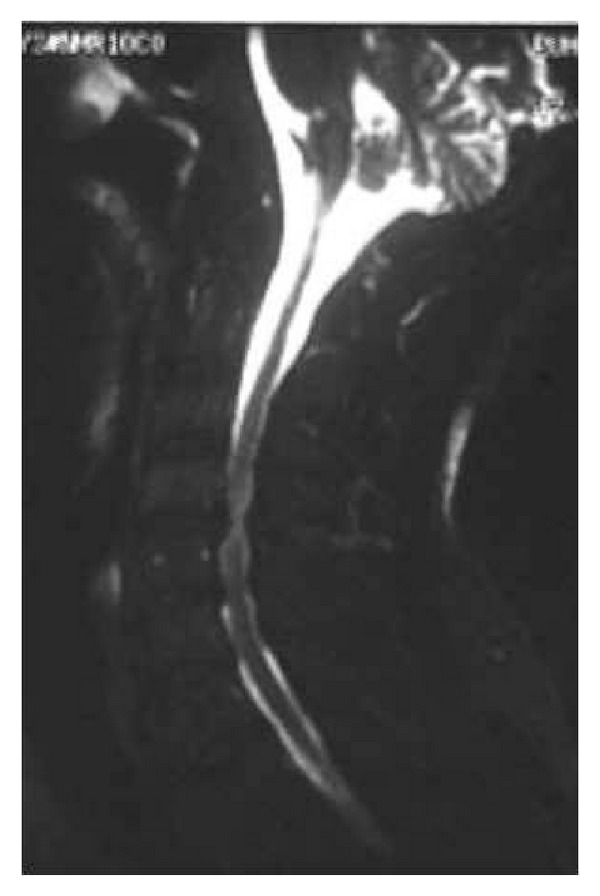
Lateral T2-weighted MRI of the cervical spine denoting significant spondylotic changes.

**Figure 3:**
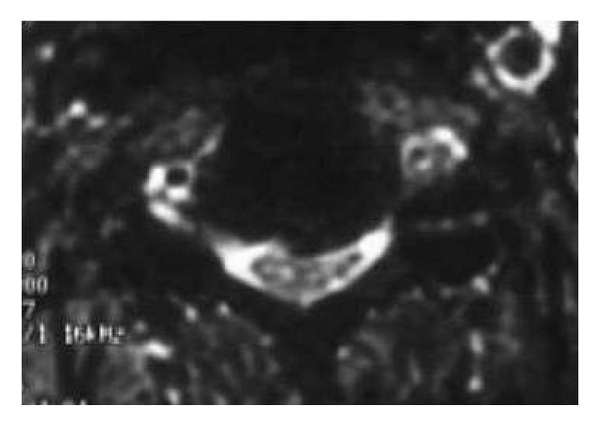
Axial T2 cervical spine denoting spondylotic changes and cord impingement.

 Several factors must be considered in selecting the appropriate patient for laminoplasty. As mentioned, cervical laminoplasty is indicated for multilevel stenosis (AP canal diameter < 13 mm) due to spondylosis or OPLL (Figures 1, 2, and 3). The procedure is generally contraindicated in kyphotic cervical pathology as there is less room for posterior drift of the cord; however, up to 10 degrees of cervical kyphosis has been shown to have acceptable results [11, 12, 14]. Further contraindications include previous posterior cervical surgery, ossification of the ligamentum flavum (OLF), and epidural fibrosis. Preservation of the posterior elements allows for reinsertion of the nuchal muscles and spinal ligaments, allowing for better preservation of lordosis. Single- or two-level stenosis may best be treated from an anterior approach.

 Although there is a resultant loss of cervical ROM, it is less incumbent than that seen with laminectomy and fusion, and therefore preservation of ROM in young patients may lead surgeons to recommend laminoplasty. If significant arthritis and/or axial neck pain is present, however, laminoplasty may not be the best option as a fusion may provide better relief through stability. Additionally, any preoperative evidence of cervical instability may be a contraindication to laminoplasty.

 A final disadvantage to laminoplasty is that nerve root decompression is more readily done successfully on the side of the open door and much more difficult to complete on the hinge side. Therefore, patients with myelopathy and bilateral radiculopathy may be better treated by other decompression options.

## 3. Surgical Technique

 Of the various techniques described for achieving decompression by means of laminoplasty, no one technique has been shown to have better results over others. The technique we employ is similar to the originally described expansive open-door of Hirabayashi and will be described here.

## 4. Room Setup/Patient Preparation

 As a posterior exposure of the cervical spine requires that patients lie prone, the anesthesia team must be experienced in managing access and endotracheal intubation in this position. Neurophysiologic monitoring should be considered for patients undergoing posterior cervical decompression. We use somatosensory-evoked potentials with care to determine baselines prior to prone positioning. Most patients receive arterial line monitoring, and we try to keep the patients mean arterial pressure at around 80–85 mm Hg to safely maintain cord perfusion.

 Once anesthesia is prepared, Mayfield tongs are applied to the patients head, the patient is transferred prone to the surgical bed, and the tongs secured to the Mayfield attachment. The neck is flexed to a position which is comfortable by the patient as demonstrated preoperatively. This limits overlap in the posterior laminae and aids in reducing the facets. We prefer to tuck the arms and tape down the shoulders to improve visualization with intraoperative radiographs; however, care must be taken not to overly stretch the brachial plexus. The bed is placed in 10–20 degrees of reverse trendelenburg to allow for improved access as well as decreased intraoperative bleeding.

## 5. Surgical Technique

 The patient is then prepped and draped in sterile fashion, the spinous processes are palpated to estimate levels, and a midline incision is made. Electrocautery is used to carry the incision deeply and expose the spinous processes, laminae and lateral masses of the desired levels, with care to preserve the facet capsules as well as the supraspinous and interspinous ligaments, as well as the interspinalis muscles. Localization can be confirmed by a lateral radiograph intraoperatively.

 The junction of the laminae with the lateral mass is identified bilaterally. The hinge is placed at this level. We prefer to place the hinge on the less symptomatic side, allowing for better decompression and easier foraminotomies of the more symptomatic side. The ligamentum flavum is taken down at the proximal and distal ends of the laminoplasty, usually C3 and C7, but left intact throughout the other levels. Using a fine tip bipolar, usually the epidural veins can be carefully coagulated as you take down the ligamentum flavum. A high speed burr is used to create a bicortical defect on the open door side just medial to the junction of the lamina and lateral mass. Completing the open side first gives the surgeon feedback as to the thickness of the lamina for preparation of the hinge side. The burr is then used to make a unicortical defect in each lamina on the hinge side. The spinous processes are tilted gently toward the hinge allowing for opening of the door, and a Kerrison rongeur is used to take down the remaining ligamentum flavum at each level.

**Figure 4:**
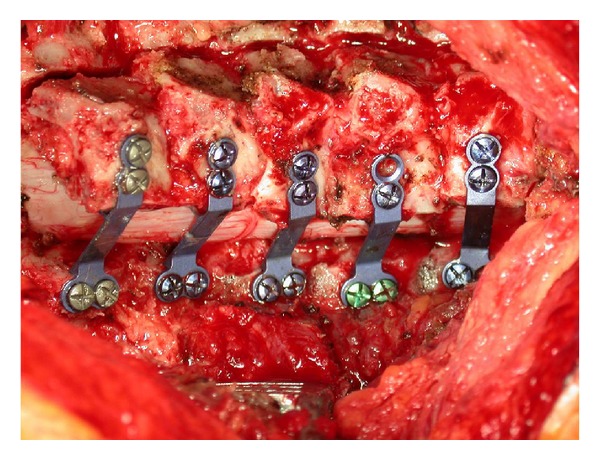
Post-laminoplasty view using plate fixation to hold the posterior hinge open.

 Fixating the door open can be done by a variety of techniques including bone block, suture, suture anchors, facial trauma plates, or laminoplasty specific plates (Figure 4). We then prefer to shorten the spinous processes with a rongeur, especially at the C6-7 level, to facilitate skin closure and decrease a postoperative prominence. The spinous processes can be shortened earlier in the procedure, though they may be helpful in opening the hinge.

**Figure 5:**
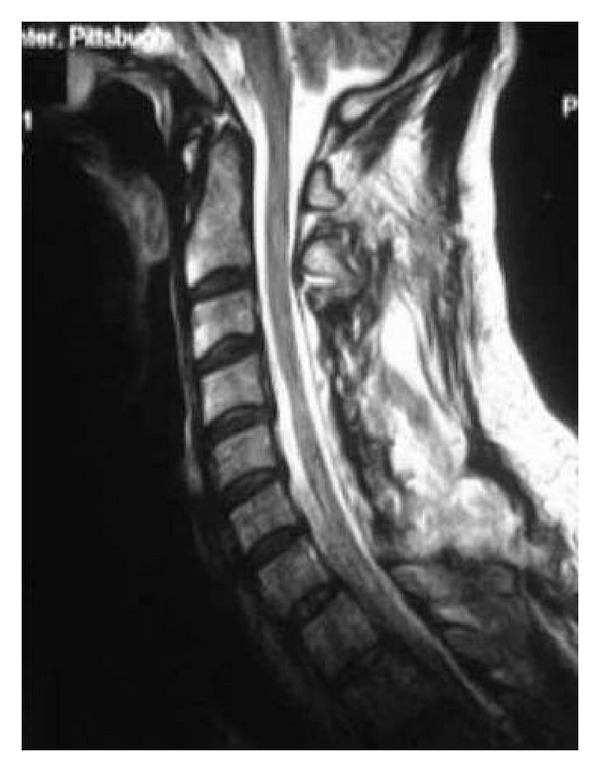
Post-laminoplasty MRI showing the space available for the cord created by the posterior decompression.

**Figure 6:**
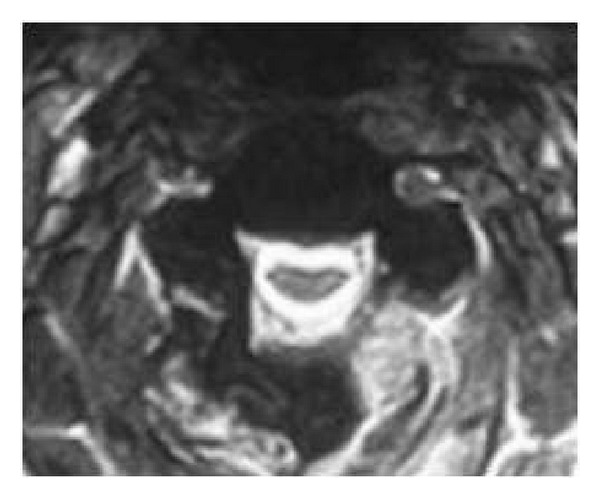
Post-laminoplasty MRI showing the open hinge and space available for the cord created by the decompression.

 A meticulous closure is done prior to leaving the operative field. We thoroughly irrigate the wound and stop all visable bleeding with cautery. A subfascial drain is placed, and the fascia is approximated with number 2 absorbable figure of eight stitches. The dermis is closed with 2–0 absorbable buried interrupted stitches, and the final skin is closed with a running subcuticular absorbable stitch. This technique should allow for adequated creating of space available for the cord (Figures 5 and 6).

 Postoperatively, we place patients in a cervical orthosis for 4 weeks. The type of orthosis, or need for one at all, is a matter of surgeon preference. A soft collar for comfort only can be appropriate, and long-term rigid bracing certainly is not required. Current evidence suggests that a shorter period of immobilization and quicker return to motion may decrease the postoperative neck discomfort and help prevent range of motion loss [15].

## 6. Outcomes

 Although it has limitations, the most comprehensive method of assessing the degree of impairment secondary to myelopathy is likely the Japanese Orthopaedic Association (JOA) score, with higher scores indicating better patient status and lower scores representing poorer patient status. Multiple studies reviewing laminoplasty have shown increases in the JOA by 55–65% [4–8]. Handa et al. [16] reported on 61 patients treated with the open-door technique which showed increase in recovery as well as JOA scores at one year. Their group was stratified by age (older versus younger than 70 years), and both groups showed improvement (62% and 59%, resp.). When cohorts are stratified by diagnosis, there is also a difference. Miyazaki and colleagues [17] reported more improvement when laminoplasty was performed for OPLL than for CSM (87% versus 76%, resp.). Interestingly, when laminoplasty was combined with a posterolateral fusion, the improvement scores for CSM surpassed those for OPLL, indicating that postoperative instability has some effect on outcomes.

 When compared with other operative techniques for CSM, laminoplasty has been shown to be as effective in relieving symptoms. Heller et al. [18] reported no statistical difference in outcomes between laminoplasty or laminectomy and fusion, but noted a 2-fold decrease in the range of motion after laminectomy and fusion. Our series found no statistical difference between laminectomy and fusion and laminoplasty; however, there was a trend toward better functional and subjective scores in the laminectomy/fusion cohort [19].

 Long-term results have further shown the effectiveness of laminoplasty. Miyazaki et al. [17] reported on patients at greater than 12-year followup and showed that the benefits of laminoplasty were maintained. Seichi et al. [20] further confirmed this in their report of 91% stability in their outcomes over 10 years in patients with CSM decompressed by laminoplasty. This was in contrast to an 81% maintenance of outcomes in patients diagnosed with OPLL decompressed with laminoplasty.

## 7. Complications

 The postoperative complications for laminoplasty are similar to those of other posterior decompression techniques. Some have advocated that there is a larger incidence of wound complications and poor healing presumably due to the increased tension created by the mass effect of elevating the posterior structures [15]. It is for this reason that we commonly debulk the more pronounced spinous processes prior to wound closure.

 Literature review of laminoplasty reveals two main issues associated with laminoplsty: nerve root palsy (specifically C5) and axial neck pain.

 A motor dominant C5 root palsy may result after laminoplasty in 5–11% of cases [10, 11, 13]. This usually occurs on post-operative day two or three and is not commonly seen immediately postoperatively. C5 is most often involved, though C6, C7, and rarely C8 root palsies have been described [15]. These motor root palsies are not unique to laminoplasty. This complication has also been reported after laminectomy and fusion or anterior decompression and fusion procedures for the same diagnoses. Sodeyama and associates reported on postlaminoplasty patients evaluated with CT myelograms who showed a mean posterior drift of 3 mm [21, 22] at the level of C5. It is hypothesized that a mechanical tethering of the nerve root in the foramina in the presence of posterior cord migration may put the C5 root under stretch and cause the palsy [23–25], though this theory does not fully explain why a C5 palsy may occur after an anterior decompression as well.

 Though range of motion may decrease by 17–50%, the loss is less than that after laminectomy and fusion [10, 11], although stiffness postlaminectomy/fusion is often downplayed as a complication as it is a goal of fusion surgery.

## 8. Summary

 Cervical spondylotic myelopathy is a progressive decline in the ability of the cervical spine to function properly. The natural history would suggest a continuous decline in neurological function which can ultimately become debilitating for patients. Current treatment theory suggests that a thorough decompression of the spinal canal can aid in preventing this decline.

 Laminoplasty, or decompression with retention of the posterior elements, offers a surgeon multiple advantages as a treatment option. The idea of a motion-sparing technique is the largest benefit when comparing laminoplasty to a laminectomy and posterior fusion. Although complications may still occur and special care must be paid to patient selection, laminoplasty is a viable option to consider when treating patients with CSM.
